# Role of the ATM-Checkpoint Kinase 2 Pathway in CDT-Mediated Apoptosis of Gingival Epithelial Cells

**DOI:** 10.1371/journal.pone.0011714

**Published:** 2010-07-23

**Authors:** Mounia Alaoui-El-Azher, Jeffrey J. Mans, Henry V. Baker, Casey Chen, Ann Progulske-Fox, Richard J. Lamont, Martin Handfield

**Affiliations:** 1 Department of Oral Biology and Center for Molecular Microbiology, College of Dentistry, University of Florida, Gainesville, Florida, United States of America; 2 Department of Molecular Genetics and Microbiology, College of Medicine, University of Florida, Gainesville, Florida, United States of America; 3 Division of Periodontology, Diagnostic Sciences and Dental Hygiene, Herman Ostrow School of Dentistry, University of Southern California, Los Angeles, California, United States of America; Health Canada, Canada

## Abstract

The cytolethal distending toxin (CDT) of the oral pathogen *Aggregatibacter actinomycetemcomitans* induces cell cycle arrest and apoptosis in various cell types. Western analysis, pharmacological inhibition and siRNA silencing were performed in human immortalized gingival keratinocytes (HIGK) to dissect the functional role of the ataxia telangiectasia mutated (ATM) pathway in the signal transduction steps triggered by the CDT. Infection of HIGK was associated with a time-dependent induction of cytoplasmic histone-associated DNA fragmentation. However, in the absence of CDT, infected HIGK underwent reversible DNA strand breaks but not apoptosis, while caspase 3 activity, p21 levels, and HIGK viability were unaffected. Caspase 9 activity was attenuated in the CDT mutant-infected HIGK compared to wild-type infected cells. Pharmacological inhibition and siRNA-silencing of the ATM downstream effector, the protein kinase checkpoint kinase 2 (Chk2), significantly impacted CDT-mediated apoptosis. Together, these findings provide insight on the specificity of the ATM-Chk2 pathway in response to the CDT of *A. actinomycetemcomitans* in oral epithelial cells, which ultimately leads to apoptosis. We further propose the existence of an unidentified factor that is distinct from the CDT, and involved with a reversible DNA fragmentation that does not trigger terminal apoptosis in oral epithelial cells. This model potentially explains conflicting reports on the biological activity of the *A. actinomycetemcomitans* CDT.

## Introduction

*Aggregatibacter actinomycetemcomitans* is the etiologic agent for localized aggressive periodontitis [1] and can also cause severe infections outside of the oral cavity, including endocarditis and brain abcesses [2] and systemic conditions such as cardiovascular disease and pregnancy complications [3], [4]. The pathogenicity of *A. actinomycetemcomitans* is influenced by both microbial and host determinants. *A. actinomycetemcomitans* produces a cytolethal distending toxin (CDT) that is part of a family of cytotoxins found in other pathogenic bacterial species such as *Campylobacter jejuni*, enteropathogenic *Escherichia coli*, *Haemophilus ducreyi* and *Shigella* species [5]–[7]. In the oral cavity, human immune cells and diverse non-lymphoid cell types exhibit variable levels of sensitivity to the CDT of *A. actinomycetemcomitans* [8], [9]. Upon an *in vitro* challenge with CDT, many cell types show a cell cycle arrest in G2, cellular distension, and ultimately cell death. In contrast, the effects of CDT on lymphocytes are apparently different and the molecular basis for an increased lymphocyte sensitivity to CDT remains moot [10], [11].

Based on sequence homology across multiple bacterial species, it has been suggested that CdtB functions as a DNase-like moiety whereby it cleaves DNA and activates the G2 cell cycle checkpoint [12], [13]. Indeed, it has been shown that purified CdtB exhibits detectable nuclease activity, although it was almost five orders of magnitude lower than that observed with control DNAse from bovine species [7]. In contrast to the popular dogma, it has been shown that CDT-induced DNA fragmentation in lymphocytes is not the result of direct effects of the toxin, but rather the irreversible effects of cell cycle arrest leading to activation of the apoptotic cascade [14]. Shenker *et al.* further proposed that the protein fold of CDT—and thus potentially its reaction mechanism—is homologous with other proteins from functionally unrelated signaling metalloenzymes, including phosphatidylinositol (PI)-5-phosphatases [7], [15]. In particular, CdtB exhibited PI-3,4,5-triphosphate (PI-3,4,5-P3) phosphatase activity similar to that of other phosphatases [7]. Furthermore, mutation analysis demonstrated that CDT toxicity correlated with phosphatase activity and that CDT-induced G2 arrest correlated with intracellular levels of PI-3,4,5-P3 in lymphocytes.

In mammalian cells, DNA damage-signaling pathways are activated following exposure to different forms of genotoxic stress, and are essential to maintain the genomic integrity and cellular viability [16]. The ATM (ataxia-telangiectasia-mutated) and ATR (ATM and Rad3-related) protein kinases play a central role in transducing DNA damage signals. Their checkpoint functions are mediated partially by the checkpoint effector kinases called checkpoint kinase 1 (Chk1) and checkpoint kinase 2 (Chk2) [17]. Activation of Chk1 and/or Chk2 causes the phosphorylation and thereby inactivation of Cdc25 (Cell division cycle 25) tyrosine phosphatases, which creates a binding site for 14-3-3 proteins and results in their export to and retention in the cytoplasm [18]. Cyclin-dependent kinase 1 (Cdk1)/Cdc2 complexes remain phosphorylated in the absence of active Cdc25 phosphatases, causing cell cycle arrest [19]. Regardless of the CDT biological activity's origin—whether related to DNA damage, phosphatase activity or both—we hypothesized that ATM may be a critical factor in the transduction pathway related to cell cycle arrest in keratinocytes. To test that hypothesis and discriminate between multiple possible downstream effector checkpoint kinases that may be involved, we used a combination of bacterial mutant analysis, phenotypic assays, pharmacological and siRNA inhibition studies.

## Materials and Methods

### Bacterial strains and Growth Conditions

*Aggregatibacter actinomycetemcomitans* strain VT1169 is a nalidixic acid and rifampicin-resistant smooth derivative from the serotype B clinical strain SUNY 465 [20]. *A. actinomycetemcomitans* strain D7S-SA is a spontaneously occurring non fimbriated (smooth) derivative from a serotype A clinical isolate strain D7S [21]. The CDT mutant strain CHE001 is an isogenic mutant in D7S-SA obtained by replacing the polycistronic operon of CDT (*cdt*ABC) with the spectinomycin cassette *aad*9 [22], [23]. *A. actinomycetemcomitans* strains were grown in Trypticase Soy Broth supplemented with 0.6% yeast extract (TSB-YE) for 24 h at 37°C in a humidified atmosphere supplemented with 10% CO_2_. When necessary, the media were supplemented with 50 µg mL^−1^ of spectinomycin, nalidixic acid or rifampicin. *P. gingivalis* strain ATCC 33277 was cultured anaerobically to mid log phase at 37°C in trypticase soy broth supplemented with yeast extract (1 mg mL ^−1^), hemin (5 µg mL ^−1^), and menadione (1 µg mL ^−1^).

### Cell culture

Human immortalized gingival keratinocytes (HIGK) [24] were maintained in keratinocyte serum-free medium (K-SFM, Gibco/Invitrogen) supplemented with 0.05 mM calcium chloride and 200 mM L-glutamine (Gibco/Invitrogen) at 37°C in a 5% CO_2_ atmosphere.

### Infection and cell treatments

For all experiments, the bacteria cultured overnight were subcultured for 3 h at 37°C (mid-log stage), harvested by centrifugation, and resuspended in antibiotic-free K-SFM media. HIGK in subconfluent monolayers (80–90% confluence) were washed with phosphate-buffered saline (PBS; Cambrex) and inoculated with bacteria in K-SFM at the multiplicity of infection (MOI) previously shown to result in every HIGK directly interacting with at least a single bacterium, determined by visual observation and confirmed by total interaction assay [25]. The baseline MOI meeting this parameter for wild type strains of *A. actinomycetemcomitans* was MOI 3000, whereas MOI 100 was used for the highly invasive *P. gingivalis*. Total interaction assays and LDH toxicity assays (data not shown) were used to optimize several factors, including degree of total interaction between bacteria and cells, duration of interaction, and the amount of host cell damage incurred by HIGK during the co-culture. The emergent treatment condition that allowed for long term studies (up to 72 hours post infection) and was thus used for all experiments, was the treatment of cells with antibiotics (gentamicin 200 µg mL^−1^) at 4 hours following the initial infection. Cell culture media changes necessary for time course studies past 48 hours also included gentamicin (200 µg mL^−1^).

In inhibition experiments, cells were pretreated for 2 h with caffeine (5 mM; Sigma), LY294002 (20 µM; Calbiochem), or Z-VAD (50 µM; Calbiochem) prior to infection with *A. actinomycetemcomitans*. After 4 h of co-culture, the cells were washed two times with PBS and the media replaced with fresh K-SFM containing gentamicin (200 µg mL^−1^) and further treated with caffeine, LY294002 or Z-VAD during the remaining time of the incubation. As a positive control for apoptosis, HIGK cells were treated with camptothecin at a final concentration of 2 µg mL^−1^.

### Lactate dehydrogenase assay

At different time points post-infection, cellular supernatants were tested for LDH release using the Cytotoxicity Detection Kit (Roche Diagnostics) and following the manufacturer's recommendation. The amount of LDH released to the supernatant (i.e., LDH leakage) was expressed as a percentage of the total cellular LDH content (i.e., sum of the LDH in the supernatant and in the cell lysate).

### Caspase 3 activity assay

After infection, HIGK were harvested, lysed on ice in sterile filtered lysis buffer for 30 min and tested for levels of caspase 3 activity using a 7-amino-4-methylcoumarin (AMC) release assay (BD Pharmingen) and following the manufacturer's protocol. Lysis buffer composition was according to the Ac-DEVD-AMC protocol, consisting of 10 mM Tris-HCL, 10 mM NaH_2_PO_4_/NaHPO_4_, pH 7.5, 130 mM NaCl, 1% Triton-X-100, and 10 mM sodium pyrophosphate. Protein concentrations were determined in parallel by a Coomassie Plus Assay Reagent (Pierce Chemicals).

### Caspase 9 activity assay

After infection in 6-well plates, HIGK were assayed using the Caspase-9 Colorimetric Activity Assay Kit (Millipore), and according to the manufacturer's instructions. Briefly, cells were lysed on ice with 500 µL chilled 1x lysis buffer for 10 minutes, harvested and transferred to a 1.5 mL tube for centrifugation. 50 µL of each clarified sample was assayed in a 96 well plate by mixture with the appropriate volumes of assay buffer, substrate, and water. Plates were read at 405 nm, compared to pNA standard curves, and were reproducible. Caspase 9 was normalized to total protein amounts based on the Coomassie Plus Assay (Pierce).

### DNA fragmentation assay

Inter-nucleosomal DNA fragmentation was quantitatively assayed by antibody-mediated capture and detection of cytoplasmic histone-DNA complexes using a Cell Death Detection ELISA kit (Roche Diagnostics).

### Protein extraction and Western blot analysis

Following infection, HIGK were recovered and resuspended in 30 µL of RIPA buffer (Santa Cruz) supplemented with protease inhibitors, 1 mM phenylmethylsulphonyl fluoride (PMSF), 50 mM sodium fluoride and 1 mM sodium orthovanadate. Protein extraction was performed at 4°C for 30 min. The protein content of the lysates was measured using a Bicinchoninic Acid Assay Kit (Pierce). Per sample, 30 µg of protein was subjected to 6–10% SDS-PAGE under reducing conditions. After electrophoresis, proteins were transferred onto PVDF-Plus membranes (Osmonics) and blocked for 1 h in Tris-buffered saline (TBS) containing 5% non-fat milk and 0.1% Tween 20. Blots were probed with the appropriate primary antibodies and peroxidase-conjugated goat anti-mouse or goat anti-rabbit secondary antibodies (1∶2000) (Santa Cruz Biotechnology). Specific signals were developed using the ECL-Plus system (Amersham). Blots were stripped with Restore Western Blot Stripping Buffer (Pierce) and re-probed with a control β-actin antibody (Santa Cruz Biotechnology). Densitometry was performed using the Kodak 1D Image Analysis Software v.3.6.1.

### Transfection of siRNA

The ON-TARGETplus SMARTpool siRNA against checkpoint kinase 2 (Chk2;GenBank Accession No. NM007194, NM145862; catalog No. L-003256-00) and the non-specific negative control non-targeting siRNA #2 (catalog No. D-001210-02-05) used in this study were obtained from Dharmacon. HIGK were seeded in 6-well-plates 20 h before transfection. The transfection with siRNA was carried out using Oligofectamine following the manufacturer's instructions (Invitrogen). Transfected cells were rinsed 24 h post transfection with PBS and maintained in culture medium until further treatment.

### Statistical Analysis

The results are reported as the mean ± SD. Statistical evaluation was performed using an unpaired, two tailed Student's- t test.

## Results

### Cytolethal Effects of *A. actinomycetemcomitans* on HIGK Requires CDT

**Figure 1:**
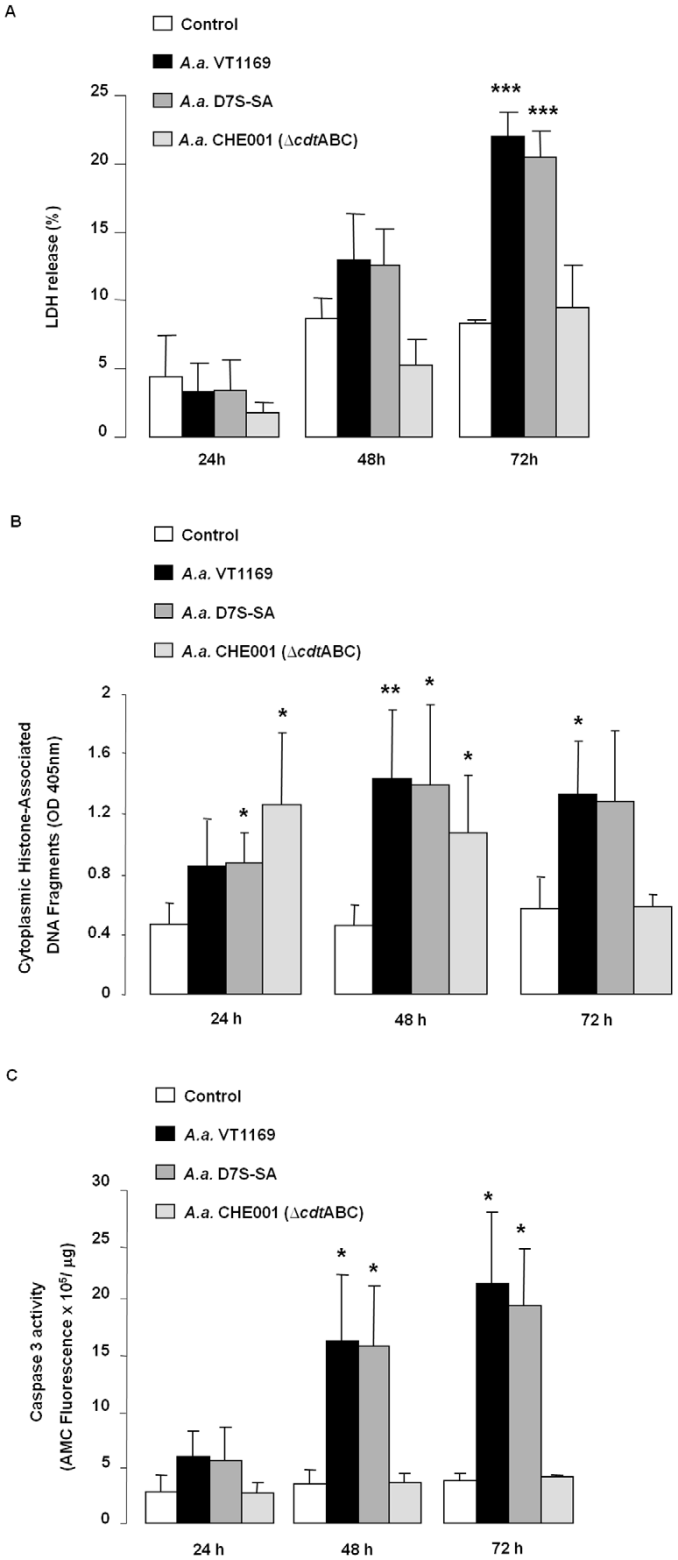
Effects of CDT on LDH release (A), DNA fragmentation (B), and caspase 3 activity (C). HIGK were plated 48 h before infection. After 4 hours of co-culture with *A. actinomycetemcomitans* strains VT1169, D7S-SA or CHE001 (Δ*cdt*ABC) at a MOI of 3000:1 or untreated, cells were washed and incubated for an additional 20, 44, and 68 h with gentamicin (200 µg mL^−1^) to obtain 24, 48, and 72 h time course data. Parameters were measured as detailed in Materials and Methods. The results are expressed as the mean ± SD of three independent experiments. *p<0.05; **p<0.01; ***p<0.001 versus untreated controls.

To investigate the toxic effects of the *A. actinomycetemcomitans* CDT on oral epithelial cells, HIGK were challenged with serotype B strain VT1169, serotype A strain D7S-SA, or CHE001 (an isogenic mutant for CDT in D7S-SA deficient in all three subunits of the CDT toxin) to confirm that the HIGK cytotoxicity was related to the expression of the CDT and was not serotype-specific. After a brief pulse with live bacteria, toxicity was indirectly measured by a lactate dehydrogenase release (LDH) assay following a time course experiment. This enzyme is exclusively intracellular and is released into the culture supernatant upon disruption of the plasma membrane integrity, as a result of apoptosis or necrosis. As shown in Figure 1A, wild-type strains VT1169 and D7S-SA induced a time-dependent release of LDH, which was characterized by a detectable toxicity at 48 hours, and a significant increase at 72 hours. LDH release was not induced above background levels by the CDT mutant strain, suggesting that cytotoxicity was CDT-dependent in HIGK.

DNA fragmentation has often been used as a direct measurement of the final steps leading to apoptosis. HIGK pulsed with two serotypes of *A. actinomycetemcomitans* showed a similar and time-dependent induction pattern of DNA fragmentation (Fig. 1B), which provided direct evidence of apoptosis induced by two different wild-type organisms. Like the caspase 3-induction pattern, DNA fragmentation was detectable at 24 hours post-infection with both serotypes and sustained over time. Unexpectedly, pulsing HIGK with the CDT mutant strain induced significant levels of DNA fragmentation at 24 hours, which was reversible in this cell population, and returned to background levels after 72 hours (Fig. 1B). The transient and reversible nature of this effect suggested that a bacterial factor distinct from CDT was capable of inducing DNA fragmentation, and yet failed to induce appreciable levels of overall toxicity or to induce terminal apoptosis. Alternatively, we cannot rule out that the conditions used herein may have selected for a sub-population of cells that did not undergo DNA fragmentation and consequently outgrew the apoptotic cell population after multiple cell divisions. Nevertheless, the irreversible cytolethal effect of *A. actinomycetemcomitans* on HIGK was consistently CDT-dependent.

A commonly used biochemical marker of apoptotic cell death is the induction of caspase 3. Since caspase 3 is an “executioner” caspase and is a reliable marker for impending apoptosis, we decided to evaluate the levels of caspase 3 in HIGK cells infected by *A. actinomycetemcomitans* deficient in the CDT compared to the wild-type infection. In line with the cytotoxicity (LDH) results presented above, caspase 3 activation was detectable earlier (at 24 hours) in HIGK exposed to both wild-type strains of *A. actinomycetemcomitans* and the activity steadily increased in a time-dependent manner, reaching a plateau after 72 hours (Fig. 1C). A dose-dependent increase of caspase 3 activity was also observed in HIGK exposed to different multiplicities of infection (MOI) by the wild-type strain VT1169 ranging from 500 to 10,000 although caspase 3 levels remained significantly elevated at all MOIs tested (data not shown). In contrast, the CDT isogenic mutant strain did not induce caspase 3 above background levels (Fig. 1C). This further suggested that the caspase 3 activation was also CDT-dependent and preceded the observable cytotoxic effect of *A. actinomycetemcomitans* CDT on HIGK by 24 hours.

**Figure 2:**
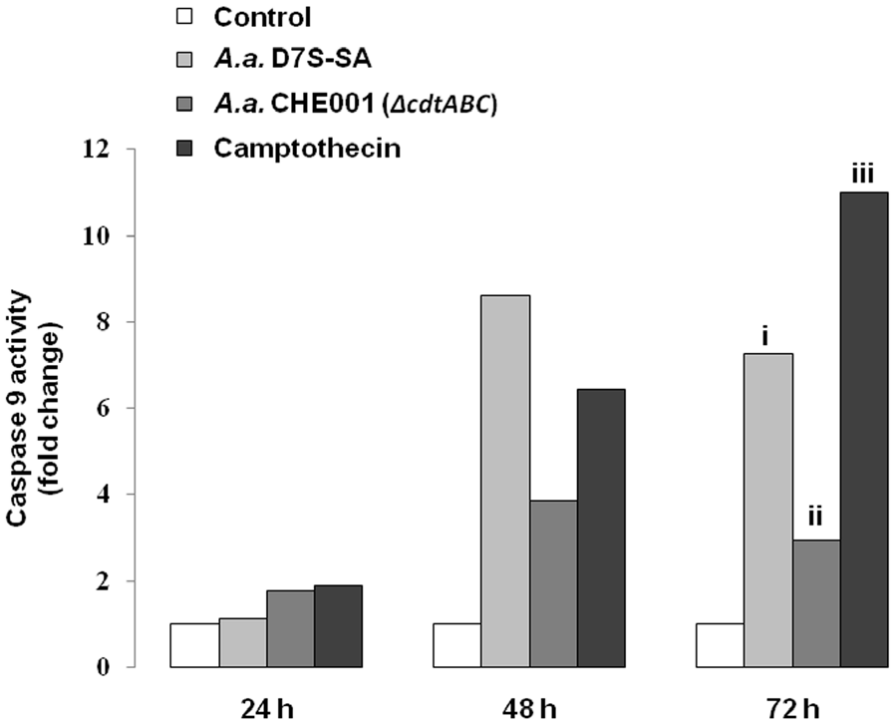
Attenuation of caspase 9 activity in cells infected by CDT^−^ *A. actinomycetemcomitans*. Cells were cultured as described in Materials and Methods and mock-infected, infected with *A. actinomycetemcomitans* strains D7S-SA, CHE001 (*Δcdt*ABC) (MOI 3000:1), or treated with camptothecin (2 µg mL^−1^). After 24, 48, and 72 hours, Caspase 9 activity was assessed for all infection conditions in duplicate and normalized to total protein. Figure is representative of two experiments. i p = 0.40 versus untreated controls; ii p = 0.89 versus untreated controls and p = 0.42 versus wild-type D7S-SA; iii p = 0.21 versus untreated controls by Student's T-Test.

In order to begin probing the pathway modulation that occurs upstream from caspase 3 in *A. actinomycetemcomitans*-induced apoptosis, we assessed the activation of caspase 9. The results of our experiments demonstrated that wild-type *A. actinomycetemcomitans* induced an increase in caspase 9 activity (Fig. 2), as observed with capase 3. In HIGK cells infected by the CDT mutant strain, caspase 9 activity was greatly attenuated, although not reduced to the baseline level of uninfected cells. This may correlate with the reversible DNA-damage also observed to occur following infection with the CDT mutant strain of *A. actinomycetemcomitans* (Fig. 1B). We are currently investigating the possible role of inhibitor of apoptosis proteins (IAPs) in this observed attenuation, which are known to negatively regulate caspases 9 and 3 [26], especially XIAP (x-linked IAP) [27]. Thus, while caspase 9 is slightly induced following infection with the *A. actinomycetemcomitans* CDT-mutant strain—perhaps in response to the partial DNA damage also observed—the damage is compensated for and indeed repaired and does not result in caspase 3 activation as is observed with wild-type infection.

### CDT-Induced Apoptosis channels through the ATM pathway

**Figure 3:**
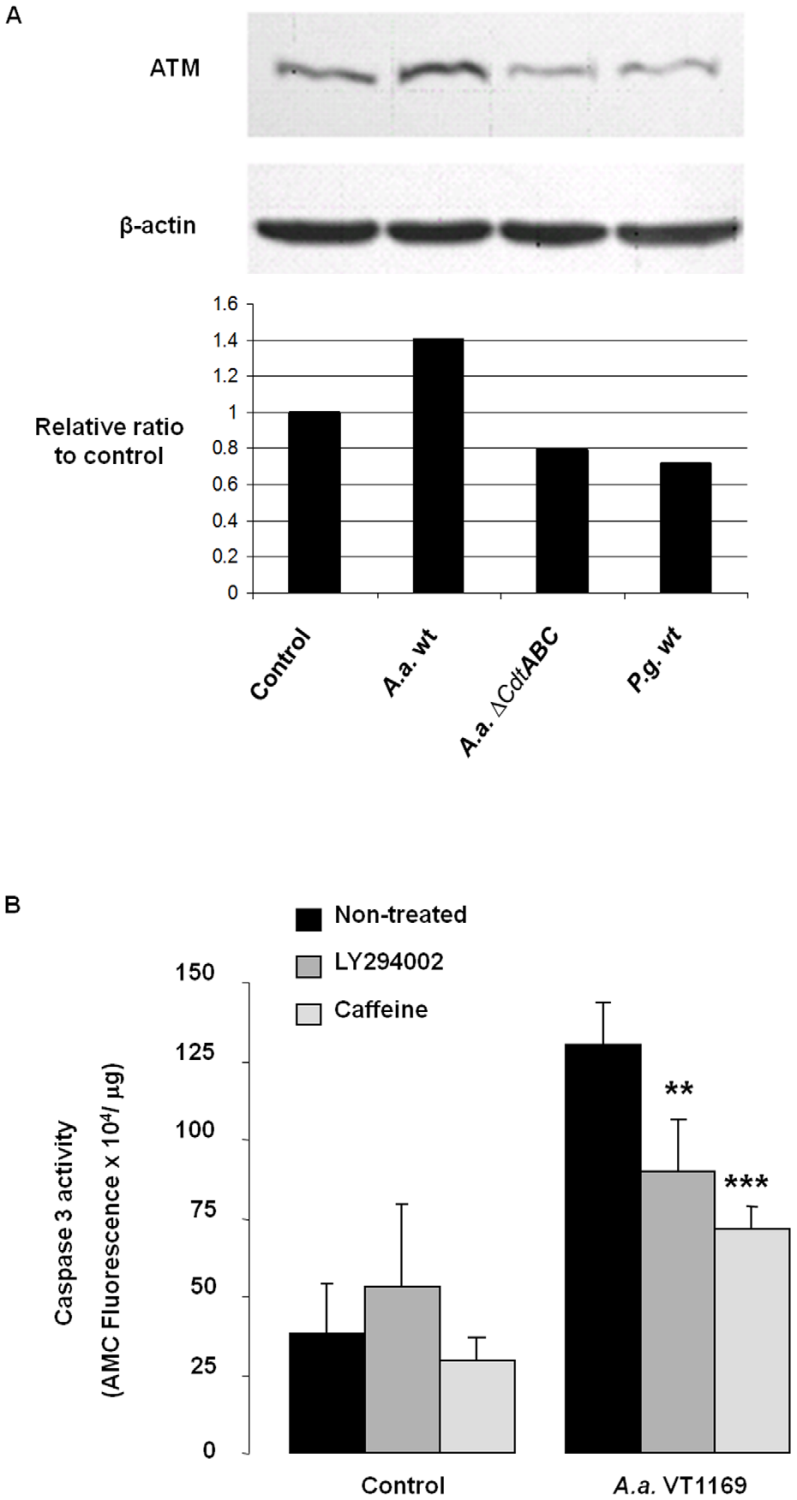
Modulation of the ATM pathway in CDT-mediated apoptosis. **Panel A.** Cells were cultured as described in Materials and Methods and infected with *A. actinomycetemcomitans* strains VT1169, CHE001 (*Δcdt*ABC) (MOI 3000:1) or *P. gingivalis* (MOI 100:1). After 24 h, Western blot was performed using antibodies for total ATM and β-actin. **Panel B.** HIGK were pretreated with or without caffeine (5 mM) or LY294002 (20 µM) for 2 h followed by incubation with *A. actinomycetemcomitans* strain VT1169 for 4 h. The cells were then washed and further incubated with gentamicin (200 µg mL^−1^) in the presence or absence of caffeine and LY294002. After a total incubation time of 48 h, cells were harvested and analyzed for caspase 3 activation. Capase 3 data presented are from two separate experiments each performed in duplicate, and combined results are expressed as the mean ± SD. **p<0.01; ***p<0.001 versus *A. actinomycetemcomitans* VT1169 infected.

We next assessed whether CDT-dependent induction of apoptosis involved signaling events mapping to the ATM pathway, as previously predicted by the transcriptional profiling of HIGK in response to a challenge with wild-type *A. actinomycetemcomitans* [25]. By Western analysis, the levels of ATM protein overexpression occurred by 24 hours following infection with the wild-type strain of *A. actinomycetemcomitans* (Fig. 3A). In contrast, the protein level of ATM remained at or below uninfected control levels in cells infected either with the CDT-mutant strain or *Porphyromonas gingivalis*, another periodontal pathogen used as a negative control (Fig. 3A). To corroborate the functional activation of the ATM pathway, caffeine and LY294002 were used as pharmacological inhibitors of the protein kinase activity of ATM [28], [29]. Both caffeine and LY294002 reduced the caspase 3 activation observed upon infection (Fig. 3B), which further supported the notion that the induction of apoptosis by CDT was ATM-dependent.

### Checkpoint protein kinase 2 is central to CDT-Induced Apoptosis

**Figure 4:**
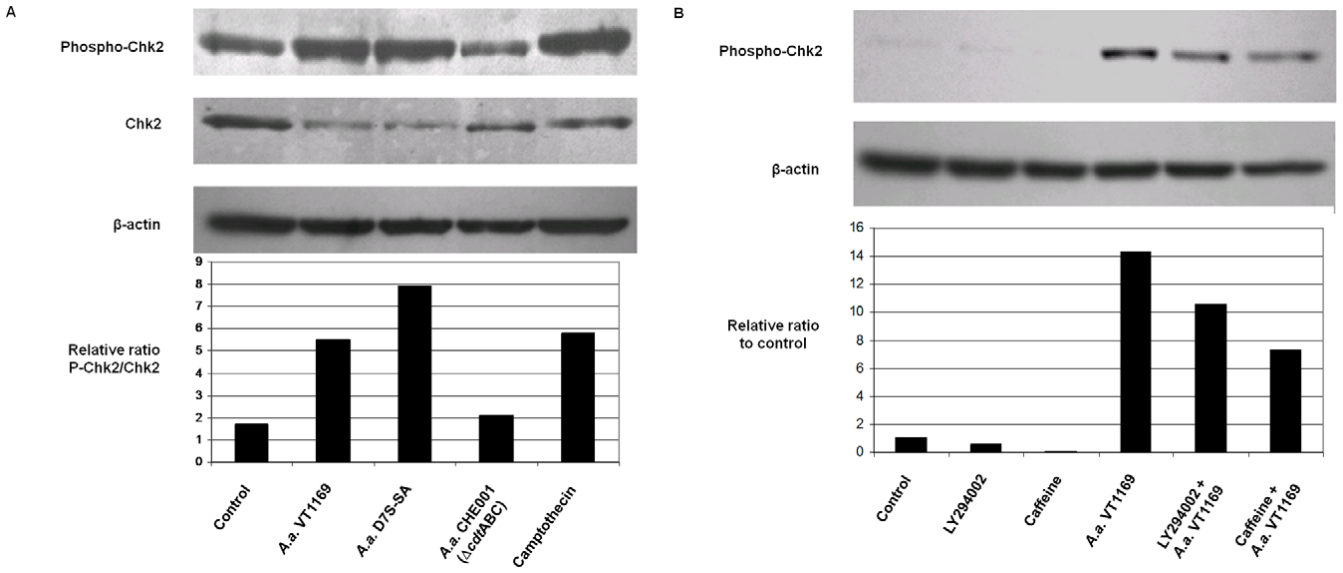
Phosphorylation and activation of Chk2 in HIGK exposed to wild type (CDT^+^) *A. actinomycetemcomitans*. **Panel A.** Cells were co-cultured with *A. actinomycetemcomitans* VT1169, D7S-SA, or CHE001 (*Δcdt*ABC) (MOI 3000:1) for 4 h, washed and incubated for an additional 20 h with gentamicin (200 µg mL^−1^), or treated with camptothecin (2 µg mL^−1^) for 4 h, as a positive control. Cell lysates were analyzed by Western blotting using antibodies specific for phospho-(Thr^68^)-Chk2, Chk2 protein and β-actin. The results are representative of two separate experiments and were analyzed by densitometry. **Panel B.** HIGK were pretreated with or without caffeine (5 mM) or LY294002 (20 µM) for 2 h followed by incubation with *A. actinomycetemcomitans* strain VT1169 for 4 h. The cells were then washed and further incubated with gentamicin (200 µg mL^−1^) in the presence or absence of caffeine and LY294002. After a total incubation time of 48 h, cells were harvested and cell lysates were prepared and analysed by Western blotting using phospho-(Thr^68^)-Chk2 and β-actin antibodies. The Western blot data are representative of two independent experiments and were analyzed by densitometry.

The next series of experiments determined whether CDT treatment could result in the activation of the checkpoint kinase 1 and/or checkpoint kinase 2, two possible downstream effectors of the ATM/ATR pathway. Uninfected HIGK expressed Chk1 (data not shown) and Chk2, as demonstrated by Western analysis (Fig. 4A), and the basal levels of expression were determined. Following *A. actinomycetemcomitans* infection, the level of Chk1 phosphorylation remained unchanged at basal levels as observed using specific antibodies against P-(Ser^345^)-Chk1 and P- (Ser^317^)-Chk1 (data not shown). However, infection with the wild-type strains VT1169 and D7S-SA of *A. actinomycetemcomitans* induced accumulation of P-Chk2 (Thr^68^) after 24 hours, while the infection by the CDT mutant strain failed to induce Chk2 phosphorylation (Fig. 4A). Chk2 phosphorylation was also observed following treatment of HIGK cells with camptothecin, a positive control for DNA damage and activation of the G2/M checkpoint (Fig 4A). It was also notable that the protein levels of Chk2 decreased upon infection with wild-type strains, but remained unchanged upon infection with the CDT mutant strain (Fig. 4A). Furthermore, the CDT-dependent phosphorylation of Chk2 was partially abolished by pretreatment with caffeine and LY294002 (Fig. 4B). Thus, upregulation of checkpoint kinase signaling resulted from the specific increase of the phosphorylation level of Chk2.

**Figure 5:**
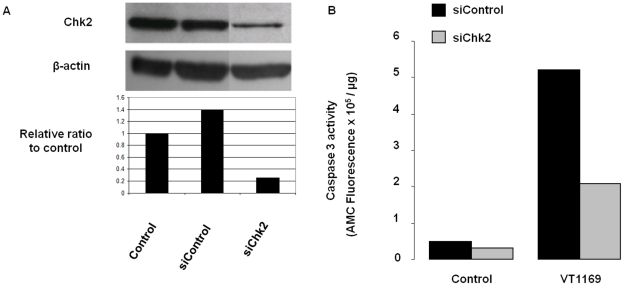
siRNA-mediated knockdown of Chk2 protein attenuates caspase 3 activation by CDT. HIGK were transfected with 40 nM Chk2 or control siRNAs for 72 h. **Panel A.** Cells were harvested and processed for Western blot analysis using Chk2 antibody to demonstrate the efficiency of Chk2 siRNA, and analyzed by densitometry. The results are representative of two separate experiments. **Panel B.** Transfected cells were co-cultured with *A. actinomycetemcomitans* VT1169 (MOI 3000:1) for 4 h, washed and incubated for an additional 48 h with gentamicin (200 µg mL^−1^). Cells were then collected and caspase 3 activity was assessed. The data are representative of three separate experiments.

To further substantiate the effect of Chk2 on the *A. actinomycetemcomitans*-CDT induced apoptosis, small-interfering RNAs (siRNA) were used to specifically downregulate Chk2 expression. HIGK were first transfected with Chk2 or control siRNAs. After 72 h of incubation, Chk2 silencing was determined by Western blot (Fig. 5A). β-actin remained unchanged, and the non-specific control siRNA did not change Chk2 expression levels. Next, we analyzed the response of siChk2-treated cells to *A. actinomycetemcomitans*-CDT-induced cell death. As shown in Fig. 5B, Chk2 knockdown greatly attenuated/prevented the activation of caspase 3 by *A. actinomycetemcomitans,* further supporting the concept that Chk2 is involved in the transduction of signals involved in apoptosis events that are induced by the *A. actinomycetemcomitans* CDT in HIGK.

## Discussion

In different bacterial species, the mechanism by which CdtB blocks mammalian cell cycling has been well established, and has shown to be mediated by DNA double strand breaks induced by a direct DNase action of CDT (as reviewed [30]). In this study, we investigated the signal transduction steps involved in the CDT-mediated apoptosis induced by *A. actinomycetemcomitans*. Previous studies disagree as to whether this organism's particular CDT directly induces DNA double strand breaks leading to cell cycle arrest and apoptosis, like most bacterial CDTs do, or whether the DNA fragmentation that is observed is the consequence of the induction of cell cycle arrest and apoptosis [12], [14], [31]. Shenker et al. proposed that CDT toxicity correlates with phosphatase activity and that CDT-induced G2 arrest correlates with intracellular levels of PI-3,4,5-P3. Under that model, indirect effect of the CDT may induce the production of endogenous DNAses converging to DNA fragmentation [7]. In the present study, live microorganisms were used to confirm that genotoxic stress can be sensed and transduced in HIGK as early as 24 hours post-infection. Furthermore, we demonstrated that the cytotoxic/apoptotic effect of *A. actinomycetemcomitans* on HIGK was exclusively CDT-dependent. Cell toxicity was assessed by LDH release and correlated with caspase 3 activation. Both phenomena followed a similar kinetic, and were CDT-dependent. Additionally, infection of HIGK with wild-type strains of two distinct serotypes induced DNA fragmentation. Furthermore, mutant analysis provided the most compelling line of evidence that supports the concept that reversible and non-lethal DNA fragmentation can be observed in the absence of CDT. Indeed, despite DNA fragmentation induction, cells infected with a CDT mutant strain were morphologically indistinguishable from control uninfected cells, whereas camptothecin-treated HIGK or HIGK infected with wild-type *A. actinomycetemcomitans* displayed clear signs of stress, damage, or overt cell death (microscopic observation, Figure S1). This is in line with earlier studies showing that the production of *Haemophilus ducreyi* CDT is crucial for cell destruction after adhesion of *H. ducreyi* to cultured cells, whereas toxin negative strains adhere but leave the cells intact [32]. One possible explanation for these findings is that cells with DNA fragmentation may have been eliminated from the culture while the surviving cells kept their ability to proliferate. The current study, however, provides evidence that in the absence of CDT, a yet unknown factor induces reversible internucleosomal DNA fragmentation without lethal cytotoxicity in HIGK. It is also possible that the antibiotic treatment used in the current study to kill the extracellular bacteria after 4 hours of incubation may account for the removal of the *A. actinomycetemcomitans* effector(s) responsible for the early DNA fragmentation, allowing the cells to recover and proliferate. Hence, the cytolethal effect of *A. actinomycetemcomitans* on HIGK seems to require CDT toxin.

Additional inhibition studies based on the pan-caspase inhibitor Z-VAD-Fmk (data not shown) supported a model whereby DNA fragmentation induced by the CDT from *A. actinomycetemcomitans* is caspase 3-dependent in HIGK. Consistently, the CDT of *A. actinomycetemcomitans* has been shown to exert its apoptotic action by activating caspases in other cell types [33], [34].

In the present study, cell death was associated with caspase 3 and 9 activation, indicating that the intrinsic mitochondria-dependent apoptotic pathway is activated by CDT. Additionally, recent studies have shown that caspase 9 is the direct target of multiple protein kinases, and the potential role of caspase 9 phosphorylation in the regulation of apoptosis is not well defined [35], [36].

Regardless of the factor responsible for the actual genotoxic stress, we demonstrated here that the genotoxic stress related to CDT is sensed by ATM and transduced by Chk2, which would result in G2/M arrest and ultimately cell death, if the DNA damage cannot be repaired. In other systems, the checkpoint functions of ATR and ATM are mediated by the checkpoint effector kinases Chk1 and Chk2, respectively [17]. These two checkpoint kinases are structurally distinct but functionally related enzymes that phosphorylate an overlapping pool of cellular substrates. Chk1 and Chk2 play a central role in transducing genotoxic stress and DNA damage signals from ATR and ATM in which Chk1 seems to be activated by ATR in response to UV-induced damage, whereas Chk2 primarily functions through ATM in response to ionizing radiation. Infection with *A. actinomycetemcomitans* did not mediate Chk1 phosphorylation, but induced a strong Chk2 phosphorylation at Thr^68^, which is the major pathway for activation of Chk2. In addition, Chk2 activation appeared to occur prior to caspase 3 activation, supporting the key role for Chk2 upstream of the caspase effectors. Pharmacological inhibition of the ATM pathway and Chk2 silencing with siRNA further supported the hypothesis that *A. actinomycetemcomitans-*induced apoptosis of HIGK follows an ATM/Chk2-dependent pathway. Because with both strategies we were able to achieve 50% reduction of apoptosis induced by *A. actinomycetemcomitans*, the involvement of other mechanisms in CDT-induced apoptosis remain to be investigated.

Although the mechanism of DNA damage-induced apoptosis remains poorly understood, the involvement of several pathways in this process have been suggested. For instance, ATM/ATR kinases can activate the tumor suppressor protein p53 that in turn either blocks cell cycle progression or induces apoptosis through regulation of the Bcl-2 family of proteins [37], [38]. In normal fibroblasts, CDT from *Haemophilus ducreyi* induced a rapid stabilization of p53 by phosphorylation, and an increased expression of the p53-regulated cyclin-dependent kinase inhibitor p21, suggesting that the checkpoint response is p-53 dependent [39]. In HS-72 cells transfected with the E6/E7 gene of the human papillomavirus type 16 (HPV-16) (resulting in the suppression of endogenous p53 function), *A. actinomycetemcomitans* CDT induced an accumulation of p21 but not p53. The cells demonstrated cell cycle arrest at the G2 stage even in the absence of active p53 [40]. The authors suggested that *A. actinomycetemcomitans* CDT-induced G2 arrest and p21 accumulation in a p53-independent pathway [40]. In contrast, cell lines that carried a nonfunctional p53, such as HeLa and HaCat, still arrested in G2 and did not up-regulate p21, indicating the involvement of a p53-independent pathway [39]. Altogether, these reports demonstrate that CDT induces a G2 block in susceptible cell lines regardless of the p53 or p21 induction.

Because HPV E6 oncoprotein has been shown to degrade p53 [41], and HIGK are known to have decreased p53 as a consequence of E6/E7 immortalization, we examined the effect of *A. actinomycetemcomitans* CDT on the expression of p21. Immunoblot analysis showed that p21 is detectable in untreated HIGK cells. Infection of cells with different strains of *A. actinomycetemcomitans* (harboring or mutated for CDT) had almost no effect on p21 expression, whereas it was upregulated in the camptothecin positive control treated cells (Figure S2). Our data suggest that other p53-independent mechanisms may account for DNA damage-induced apoptosis. In this context, DNA damage has been shown to down regulate Bcl-XL activity through deamidation, leading to apoptosis in cells deficient in p53 [42]. It has also been demonstrated that Chk2 phosphorylates the promyelocytic leukaemia (PML) protein that in turn induces apoptosis [43]. It remains unclear whether the CDT can mediate DNA fragmentation at an early time-point upon challenging oral epithelial cells with live bacteria, but it is evident that the CDT triggers events that mediate caspase activation and further precipitates the levels of irreversible DNA fragmentation, ultimately leading to apoptosis of oral epithelial cells.

In conclusion, this study promotes a better understanding of the cellular events related to cell cycle arrest and downstream consequences caused by CDTs. In a larger context, elucidating the effects of CDT upon epithelium turn-over in a number of different cell types, in conjunction with the induction of apoptosis in immune cells, may increase our understanding of aggressive periodontitis. Left untreated, CDT-affected lesions may potentiate the invasion of deeper tissue and the dissemination of bacteria through the body, which may in turn have serious consequences in the etiology of certain cardiovascular diseases and low birth weight.

## Supporting Information

Figure S1 HIGK infected by *A. actinomycetemcomitans* lacking CDT resemble control normal cells and are distinct from damaged WT-infected HIGKs. Cells were mock infected (A), co-cultured with *A. actinomycetemcomitans* D7S-SA (MOI 3000:1) (B), CHE001 (Δ*cdt*ABC) (MOI 3000:1) (C), or treated with camptothecin (2 µg mL ^−1^) (D) for 4 h, washed and incubated for an additional 68 h with gentamicin (200 µg mL^−1^). HIGK were observed 72 h post-infection microscopically at 200× magnification with a Zeiss Axiovert 25 microscope and imaged with a Canon Powershot G2 4.0 M pixel digital camera.
(5.62 MB TIF)

Figure S2 p21 is not significantly modulated in HIGK cells by *A. actinomycetemcomitans* CDT. Cells were co-cultured with *A. actinomycetemcomitans* VT1169, D7S-SA, or CHE001 (Δ*cdt*ABC) (MOI 3000:1) for 4 h, washed and incubated for an additional 20 h with gentamicin (200 µg mL^−1^), or treated with camptothecin (2 µg mL^−1^) for 4 h, as a positive control. Cell lysates were analyzed by Western blotting using antibodies specific for p-21 and GAPDH. The results are representative of multiple experiments and were analyzed by densitometry.
(0.51 MB TIF)
