# Supplementary figures and images for: Role of the ATM-Checkpoint Kinase 2 Pathway in CDT-Mediated Apoptosis of Gingival Epithelial Cells
Source: PLoS One. 2010 Jul 23;5(7):e11714. doi: 10.1371/journal.pone.0011714 (PMC2909199; doi:10.1371/journal.pone.0011714)

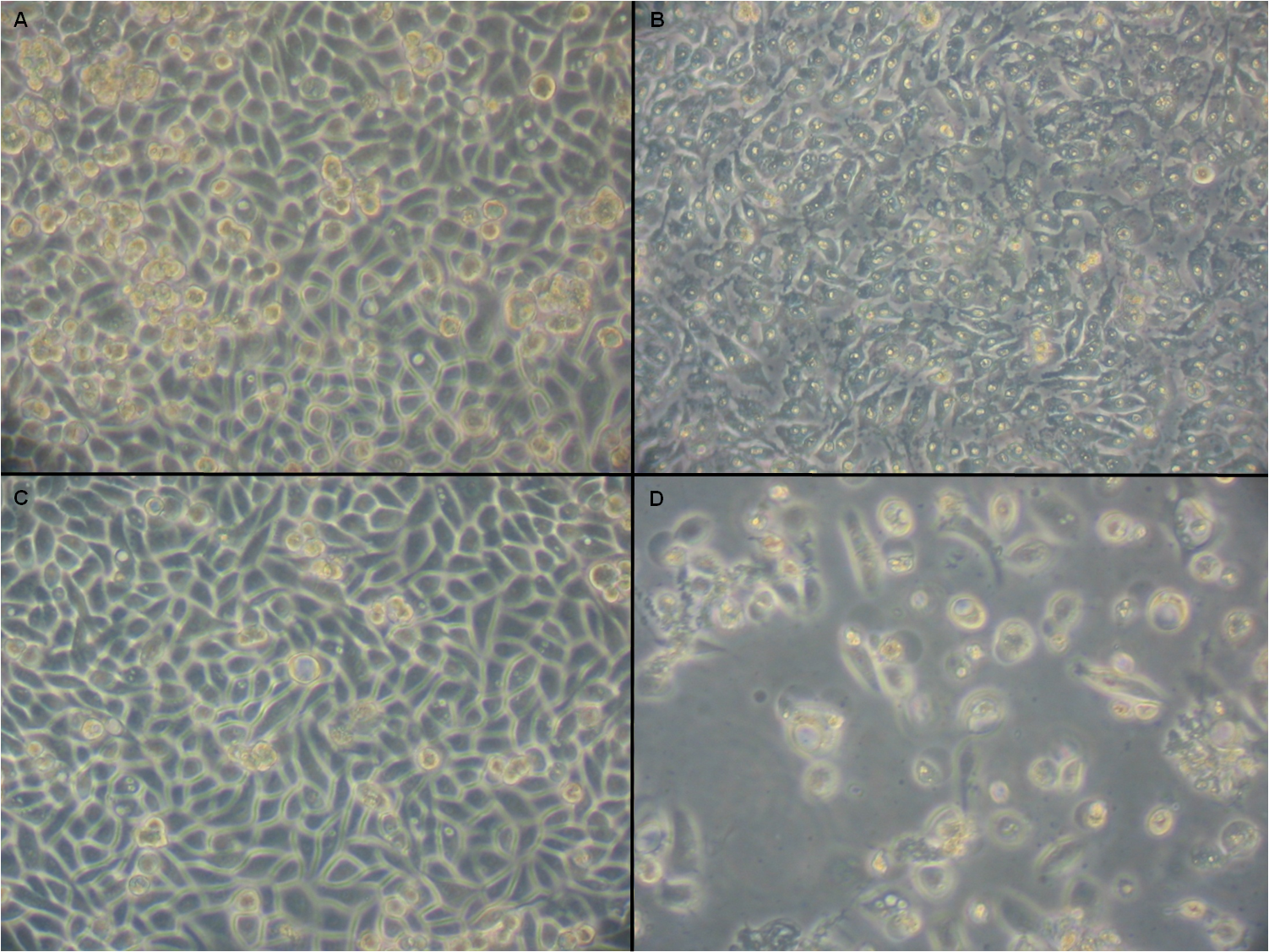

Supplement: Figure S1 — HIGK infected by A. actinomycetemcomitans lacking CDT resemble control normal cells and are distinct from damaged WT-infected HIGKs. Cells were mock infected (A), co-cultured with A. actinomycetemcomitans D7S-SA (MOI 3000:1) (B), CHE001 (ΔcdtABC) (MOI 3000:1) (C), or treated with camptothecin (2 µg mL −1) (D) for 4 h, washed and incubated for an additional 68 h with gentamicin (200 µg mL−1). HIGK were observed 72 h post-infection microscopically at 200× magnification with a Zeiss Axiovert 25 microscope and imaged with a Canon Powershot G2 4.0 M pixel digital camera. (5.62 MB TIF) [file pone.0011714.s001.tif]

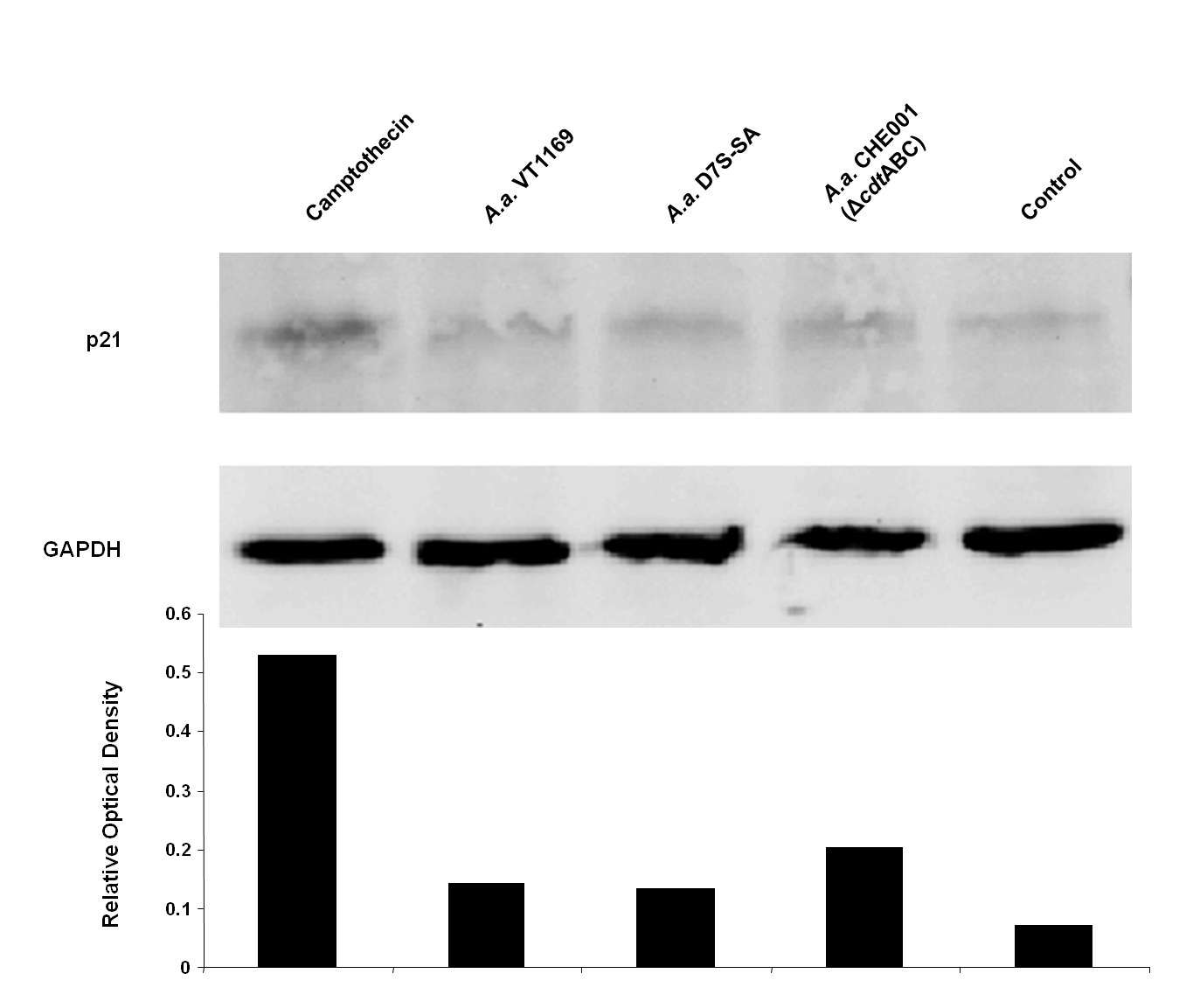

Supplement: Figure S2 — p21 is not significantly modulated in HIGK cells by A. actinomycetemcomitans CDT. Cells were co-cultured with A. actinomycetemcomitans VT1169, D7S-SA, or CHE001 (ΔcdtABC) (MOI 3000:1) for 4 h, washed and incubated for an additional 20 h with gentamicin (200 µg mL−1), or treated with camptothecin (2 µg mL−1) for 4 h, as a positive control. Cell lysates were analyzed by Western blotting using antibodies specific for p-21 and GAPDH. The results are representative of multiple experiments and were analyzed by densitometry. (0.51 MB TIF) [file pone.0011714.s002.tif]
